# Picturing Mental Health on Instagram: Insights from a Quantitative Study Using Different Content Formats

**DOI:** 10.3390/ijerph19031608

**Published:** 2022-01-30

**Authors:** Isabell Koinig

**Affiliations:** Department of Media and Communications, University of Klagenfurt, 9400 Klagenfurt, Austria; isabelle.koinig@aau.at; Tel.: +43-463-2700-1814

**Keywords:** mental health, Instagram, influencer, social support, comics

## Abstract

Background: COVID-19 has changed individual lives to the core. Through national curfews and social distancing, individuals’ social lives changed and led to increased levels of stress and mental health problems. As another consequence, communication, especially among young people, has shifted to social networking sites, where particularly young adults sought help for their mental health problems. In recent years, Instagram has received recognition for its health-enhancing potentials. On this social networking site, more than 1 billion users worldwide post 500 million stories and images per day. Methods: During COVID-19, increasing mental health incidences were reported throughout the world, and have led mental health influencers to gain in relevance. The proposed study is based on a quantitative survey. In order to test how different content formats utilized by mental health influencers —motivational quotes, comics, or influencer posts—are evaluated by Instagram users, a cross-sectional quantitative study was conducted in April 2021. Data collection was based on convenience sampling. In total, 532 Austrian respondents between 16 and 34 years of age participated in the survey. Results: Overall, the content that received the most favorable evaluations were both the motivational quote (M = 4.23) and the influencer post (M = 4.12), while the comics scored lower in terms of evaluations (M = 3.72). Conclusions: Respondents’ preference of content suggests that individuals seek out content that boosts their esteem or content with a “human touch”. Explanations as to why the selected content formats were preferred over the other formats are offered alongside a future research outlook.

## 1. Introduction

The COVID-19 pandemic has forced individuals to adapt their lives to a new normal and brought with it national curfews and social distancing. In consequence, individual stress levels rose due insecurities as well as social isolation—and all of these aspects were found to have a negative effect on people’s mental health [1], leading to increasing mental health incidences [2,3]. In fact, since the beginning of the pandemic, the psychological stress on adolescents in particular has significantly increased [4]. According to Dlugosz [5], the majority of the 18–29 year olds have experienced depression and anxiety as a result of COVID-19. Moreover, individual conversations increasingly shifted towards social networking sites [6]. With the number of social media platforms multiplying, as well as an increasing number of actors participating in public health discussions [7,8], new forms and ways of creating awareness and offering support for mental health have emerged [9]. Given the unavailability of selected services, amongst them also therapy sessions and counseling, the younger generation turned to Instagram to seek support for their mental health problems [7,10].

On this social media platform, influencers awarded them with a kind of lifeline, offering them support and an open ear in their isolated daily lives [11]. The so-called mental health influencers actively thematize issues, such as depression and anxiety, and do not only convey an understanding of the subject area, but also express empathy to those affected, encouraging discussions by posting positive quotes, pictures and even personal stories. The present contribution seeks to investigate how Instagram users evaluate different content formats associated with mental health issues. The study presented in this paper is based on a cross-sectional quantitative survey. Building on the results of a previous content analysis (the author, forthcoming), recipients’ evaluations of the selected content formats (motivational quote, comics and influencer post) were investigated and critically reflected upon. While textual content plays a core role in assessing the post’s quality [12], it is, nonetheless, not sufficient to look at content alone. Instead, it also seems to be worthwhile to investigate how Instagram users react to different content formats and whether these posts do indeed award them some form of (online) support. The focus of the analysis is on how social support offered by mental health influencers can involve recipients more actively in their health.

## 2. Theoretical Background

### 2.1. Health Communication on Social Media 

To date, the largest part of research in health communication focused on the negative consequences of excessive social media use [13], including increased rates of suicidality, loneliness and low empathy [14]. Moreover, social media has been found to contribute to negative mental health outcomes across a variety of platforms, including Facebook, Twitter, YouTube and Instagram [15]. Instagram, in particular, has been criticized for enabling “public health surveillance” [16] and has been associated with several negative health effects resulting from passive media consumption, such as social comparison [17], loneliness [18], (cyber)bullying [19] and mental health issues [18].

Lately, however, academic studies have also started to recognize social media for its health-enhancing purposes, given that social media’s effect on mental health has been inconclusive [14] and even weakened by more recent studies [20,21,22,23,24]. This has led Instagram—amongst other social media outlets—to be regarded as a platform that individuals can use to enhance their health [25,26]. For instance, Instagram users can inspire others to follow their examples, leading to higher levels of well-being [27], or allow individuals to connect with like-minded people. Studies have also uncovered that social media messages can help to reduce mental health issues and the stigmatization associated therewith [28].

Recently, health communication is also increasingly taking place on Instagram. With more than 1 billion users worldwide, and 500 million stories posted per day [29], this platform’s interactive visual set-up does not only allow for the utilization of different content formats, but has also led influencers to gain in relevance. In general, influencers are defined as “online personalities with large numbers of followers, across one or more social media platforms (e.g., YouTube, Instagram, Snapchat, or personal blogs), who have an influence on their followers” [30,31], [32] (p. 58). Often compared to spokespersons and celebrities, influencers get in touch with a large number of people at the same time [12]. Their messages are usually characterized by a high degree of relatability and enjoy high levels of trust [33]. As Instagram offers a variety of message formats, influencers’ posts usually have both an informational and entertainment value [33]. 

### 2.2. Mental Health Influencers and Online Social Support 

During the COVID-19 pandemic, mental health influencers were ascribed a special role. As some services were not available, for instance, therapy sessions and counseling, the younger generation instead turned to Instagram to seek support for their mental health problems [7,34,35,36]. On this social media platform, influencers awarded them with a kind of lifeline, offering them support and an open ear in their isolated daily lives [37,38]. 

On social media, diverse forms of peer support are offered [39,40,41]. Consequently, social networks become places where individuals can provide as well as receive hope, advice and reflection [7,37,42]. According to Chang [43], four forms of support can be distinguished:

(1) *Informational support*: respondents are provided with information on proper treatment, medication or access to providers;
(2) *Esteem support*: individuals receive positive comments and words of encouragement;
(3) *Emotional support*: other social media users express empathy, support and understanding for individuals’ problems, hardships and struggles, also offering hope and guidance;
(4) *Network support*: affected social media users (both influencers and followers) share personal recounts of their experiences, e.g., mental health struggles.

In 2020, authenticity was identified as one of the top trends on Instagram. Building on the concept of inspiration, influencers more frequently started to portray themselves in the form of their authentic selves (as part of a more holistic approach [44]), which can elevate their standing and credibility amongst their followers. Influencers thus started to thematize their personal mental health struggles, utilizing the diverse and varied presentation modes available on Instagram, which can increase both the visibility and relevance of the mental health discussion on social media.

### 2.3. Different Content Formats on Instagram 

Instagram is principally a mobile application that invites users to capture the mundane moments in their everyday lives and sometimes share intimate details of their lives [45]. On Instagram, influencers can choose from a variety of content formats. The following three formats were selected based on a previously conducted content analysis (the author, forthcoming).

#### 2.3.1. Motivational Quote 

Motivational quotes present a form of informative content. According to previous studies, informative content is particularly suited to influence individuals to change their behaviors, while also being able to induce changes in others [46]. Following the example of Dodds and Chamberlain [47], who looked at how Brazilian nutritionists presented their food arguments online; “motivational posts” refer to influencer statements through which influencers tried to give the power of control to their followers. For instance, motivational statements are employed to achieve positive changes in life as a whole. At this point, influencers would talk about life-changing incidents, in which improving mental health is seen to be up to the individual and their choices, respectively. Their recounts (also referred to as “compassionate quotes” [48]) almost seem like those from a psychologist or mental health care provider. As such, this motivation is not based on fear, but on individual empowerment, i.e., individuals are seen as having the power to change their own life. Previous studies confirmed that 72% of social media users seek support from others who share their experiences [49], but also like to be encouraged to become the agents in their own treatment [50].

#### 2.3.2. Comic

Comics present one “natural and capable” medium of involving individuals more strongly in health-related decision making [51]. Comics are usually characterized by a simple composition and schematic representation, which can aid comprehension and invite individuals to identify with the narrative or story developed in the comic [52,53]. Moreover, the visuality of comics makes abstract concepts more concrete [54] and aids memorization [55]. In terms of processing, comics require dual coding, whereby information is retrieved both visually and textually [56]. While this dual presentation holds the potential to involve individuals more strongly in health-related decision making [51,57], it also facilitates processing, as the heuristics (i.e., mental shortcuts) activated during information retrieval can induce individuals to change their behaviors [58,59].

The potentials of comics have already been discussed in a variety of studies, some of which have also been conducted in the health communication context. Although their potential to induce behavioral changes is often neglected [57,60], comics’ “psychological and cognitive effects of embodiment and narrative (can translate) lived experiences into narratives” [61] (p. 35), an aspect that has been found to benefit both anti-stigma communication and mental health communication [56,62,63].

#### 2.3.3. Influencer Post

Mental health influencers need to be perceived as individuals who are capable of informally influencing the attitudes or behaviors of their followers [64]. Even though the “dawn of the selfie era” has been announced [65], according to WordStream [66], of the approximately 40 billion photos posted on Instagram, only 282 million (or 0.7%) are selfies. Images featuring faces are not only capable of easily grabbing attention but can also readily convey emotions [65]. In selfies, influencers portray themselves, and posts featuring influencers can benefit from the credibility associated with their persona. Influencers are usually known to the public and their identification is based upon elements, such as admiration, association, aspiration or recognition [67]. The credibility of a celebrity endorser positively impacts the credibility of the endorsed content [68], since source credibility is also dependent upon the quality of the argument and persuasive strength of the endorser.

Having introduced the most prominent content formats utilized on Instagram, the study presented herein intends to answer the following research question:

*RQ: Which content format as utilized by mental health influencers is evaluated most favorably by Austrian respondents in both (a) affective and (b) cognitive terms?*

## 3. Empirical Study

### 3.1. Study Purpose 

The present contribution pays tribute to the fact that mental health influencers employ different content formats on social networking sites, including Instagram. To this end, we seek to investigate how recipients evaluate different post formats by mental health influencers on Instagram. Based on the findings of a previously conducted content analysis on the design of mental health influencer posts (the author, forthcoming), the cross-sectional quantitative survey sets out to determine which content format receives the most favorable evaluation by Instagram users, both in cognitive and affective terms. For this purpose, the three different message formats previously presented were selected as stimuli: (1) a motivational quote, (2) a comic or (3) a post featuring a mental health influencer. The three stimuli are actual posts by self-identified mental health influencers and were chosen since they constitute the three dominant formats as identified by the content analysis, but also correspond with different forms of social support [43].

(1) Stimulus 1 was a *motivational quote*: “Your bad days do not define you”. The quote is featured in capital letters against a floral, pastel-colored background. The motivational quote served as an expression of emotional support, for it is based on expressions of empathy and caring.
(2) Stimulus 2 contained a *comic* that was made up of three different frames. The visual featured an illustration of a ghost walking towards a cave. The illustration was accompanied by several textual sequences that read as follows: “Sometimes we just need to cry out. And that’s ok”. Given that it is based on a (informative) statement and on factual information that can assist individuals in dealing with their mental health problems, this post type classifies as informational support.
(3) Stimulus 3 was an *influencer post* and depicted a mental health influencer posing next to a positive statement (“You are beautiful”) on a restaurant’s mosaic bathroom floor. Since the influencer is featured in the visual herself and creates a connection through companionship, this post qualifies as network support.

After investigating the relevance individuals attribute to Instagram as a communication platform, we asked some general questions regarding their communication and posting behaviors on Instagram. Afterwards, their previous experiences and the social support as received from other Instagram users were scrutinized, before the respondents were exposed to the three stimuli. After reading through each post, questions related to message evaluation were posed. The questionnaire concluded with some questions on mental well-being as well as demographic information.

### 3.2. Study Population and Description 

In order to test how different content is evaluated by Instagram users, a cross-sectional quantitative study was conducted. The participants were deemed suitable to participate in the study if they were active Instagram users. This particular approach was chosen since it is suited to work out specific characteristics that exist in an online community. 

The quantitative online survey was carried out via Lime Survey. Utilizing convenience sampling, the invitations for participation in the study were sent out through social media platforms by students participating in a quantitative method class at a Central European university. The survey took place in April 2021. The initial survey sample consisted of 604 respondents. In a first step, all respondents that had indicated to not be active on Instagram (as determined through a filter question) were excluded. Moreover, the data was checked for answer patterns (e.g., flatliners, inconsistent answers and short answering periods). Ultimately, the total sample upon which the analysis is based consisted of 532 respondents.

In terms of age, the respondents were between 16 and 34 years old, with an average age of 23.9 years. While the study is based on a non-student convenience sample, the target group’s average age corresponds with that of the Instagram user base [69,70]. With regard to gender distribution, the largest part of the sample was made up of women (f = 65.7%; m = 34.3%), who are renowned to be more invested in health-related matters [71] and also constitute the majority of Instagram users [69,70].

### 3.3. Operationalization of Variables 

A structured questionnaire was utilized. All items included in the questionnaire were derived from established scales, which were translated into German. Answers to each question were reported on a 7-point Likert scale ranging from (1) “I do not agree at all” to (7) “I fully agree”. Factor analyses revealed the items of all multi-item variables to load on one single factor (First, we conducted the Kaiser–Meyer–Olkin (KMO) test to determine how suited items are for factorization. In a second step, we calculated Cronbach’s alpha values, which determined the internal consistency or average correlation of the items used in the questionnaire to gauge the reliability of the questionnaire); Finally, the items were combined for analysis.

- *Individuals’ Attitudes towards Instagram* were measured with two items adapted from the Facebook Intensity Scale [72] (KMO = 0.500, *p* = 0.000; alpha = 0.855).
- *Individuals’ Instagram Use* was reported via one question adapted from Ellison et al. [72] that read as follows: Instagram is part of my everyday activity.
- *Individuals’ Daily Instagram Routine* was determined via a single item, following the example of the Facebook Intensity Scale [72]: In the past week, how many hours did you spend on average actively using Instagram?
- *Individuals’ Experiences on Instagram* were established via 3 items of the Scale of Positive and Negative Experience [73] (KMO = 0.689, *p* = 0.000; alpha = 0.776).
- *Individuals’ Social Media Integration* (as based on the Social Media Integration Scale [74,75]) was measured via four items (KMO = 0.754, *p* = 0.000; alpha = 0.803).
- *Individuals’ Mental Health State* was determined through the utilization of the Flourishing Scale [76], which tested for respondents’ emotional well-being. It consisted of 5 items (KMO = 0.798, *p* = 0.000; alpha = 0.757).
- *Social Comparison on Instagram* was measured via 2 items adopted from Schneider and Schupp [77] (KMO = 0.500, *p* = 0.000; alpha = 0.701).
- *The Social Support as received from other Instagram Users* was based on the Online Social Support Scale [78]. It was reported with 4 items (KMO = 0.811, *p* = 0.000; alpha = 0.876).
- *Cognitive Post Evaluation* was measured with 3 questions adapted from MacKenzie and Lutz [79] (KMO = 0.708, *p* = 0.000; α = 0.832).
- *Affective Post Evaluation* was established through 5 items from the same scale [79] (KMO = 0.854, *p* = 0.000; α = 0.891).

## 4. Results

The present study was concerned with scrutinizing which message format was received most favorably by Instagram users. In a first step, individuals’ Instagram usage habits were assessed, before responses to the different content formats were determined. In general, results indicate that individuals hold positive attitudes towards Instagram (M = 5.0836; SD = 1.30263). The majority also seems to regard Instagram as part of their daily routines (M = 5.75, SD = 1.803), whereby the average use amounted to approximately 2 hours per day (SD = 4.4). Respondents further indicated to have had predominantly positive experiences of Instagram in the past (M = 4.8935, SD = 1.24535). 

These general results then seem to suggest that social media plays an essential part in individuals’ lives. Nonetheless, study findings do not yet support the notion that Instagram has started to replace traditional face-to-face relationships with others, as respondents state not to solely rely on Instagram to build and maintain social relations (M = 3.6631, SD = 1.45251).

Previous studies have attested individuals with mental health problems to turn to the Instagram community for advice and support [43]. Respondents claimed to be quite satisfied with their lives overall, which, if reverse-coded, insinuates that they do not suffer from mental health problems (M = 5.5816, SD = 1.07708). Nonetheless, when we investigated whether they compared themselves to other Instagram users, the majority indicated to do so (M = 5.4746; SD = 1.38080). Fewer respondents attributed relevance to Instagram as a platform for social support (M = 3.3040, SD = 1.56799). 

In a second step, the individuals’ responses towards the three different content formats were investigated, which all corresponded with different forms of social support. All posts contained only a limited amount of text and, for this reason, the respondents’ evaluations of the posts in cognitive terms were below average (M_MQ_ = 3.2340; M_C_ = 3.2054; M_INF_ = 3.2016). Evaluations did not differ significantly when taking content format—and with it, support type—into account (T = 0.451, *p* = 0.652). When splitting the results by gender, females evaluated all content formats significantly higher than males: motivational quote (M_f_ = 3.5192; M_m_ = 2.4507; F = 46.888, *p* = 0.000), comic (M_f_ = 3.4538; M_m_ = 2.5229; F = 29.939, *p* = 0.000) and influencer post (M_f_ = 3.4590; M_m_ = 2.4947; F = 35.916, *p* = 0.000). In terms of affective evaluations, which explored the emotions triggered in respondents during message reception, the motivational quote (*emotional support*; M = 4.2293) and the mental health influencer post (*network support*; M = 4.1231) outperformed the comic (*informational support*; M = 3.7209). Again, the results significantly differed when taking the respondents’ gender into account: motivational quote (M_f_ = 4.6160; M_m_ = 3.1673; F = 87.827, *p* = 0.000), comic (M_f_ = 3.9788; M_m_ = 3.0123; F = 34.873, *p* = 0.000) and influencer post (M_f_ = 4.4179; M_m_ = 3.3134; F = 42.304, *p* = 0.000). When comparing the scores, highly significant differences in the affective evaluations were reported for the motivational quote/comic comparison (T = 7.392, *p* = 0.000) and the influencer/comic comparison (T = 4.950, *p* = 0.000). Evaluations did not significantly differ between the motivational quote and influencer post (T = 1.429, *p* = 0.000).

## 5. Discussion and Implications

The present study was based on a quantitative survey that set out to test respondents’ evaluations of different content formats as posted by mental health influencers on Instagram. The study results suggest that content—regardless of its format—was seen as not particularly cognitively stimulating, while affective post evaluations were slightly better, yet merely above the scale’s midpoint. The findings might be explained as follows: respondents indicated to spend only a limited amount of time on social media per day, and also not to turn social media for online support. This might insinuate that individuals selectively choose the content they consume, or rather they consume social media for entertainment purposes but do not seek advice or guidance. This finding is in line with previous research results [80,81].

The research question on which this study was based scrutinized which of three prominent content formats—i.e., the motivational quote, the comic or the influencer post—were most favorably received by the respondents. In terms of content, interestingly, neither the motivational quote (*emotional support*) nor the influencer (*network support*) received the least favorable evaluations, unlike the comic (*informational support*). These results contradict previous research findings that attest the ability of comics to improve the involvement with health information [82] through character identification [51] and “narratives of lived experiences” [61]. More positive evaluations of the influencer post suggest that pictures that tell stories (and feature influencers) allow the affected individuals to deal better with their experiences and increase the acceptance of mental health issues [63]. Preference of format further suggests that individuals seek content that boosts their esteem or content with a “human touch”—results that are also in line with previous research findings: Pettigrew and colleagues [35] found that a spokesperson who possessed considerable knowledge on the topic was considered as an ideal candidate to speak up about health issues. Nonetheless, a more thorough investigation into what influences individuals’ content selection is needed.

An additional reason as to why comics were perceived as less favorable when compared to motivational quotes and influencer posts might be explained by dual coding [51,56]. Following the limited cognitive capacity model [83], also referred to as the limited cognitive capacity model of information processing, the processing of stimuli requires mental resources, and humans only have limited mental resources at their disposal. Messages with arousing or more complex content (such as comics, which are made up of multiple frames and consist of visual and textual elements) require a more cognitive capacity to process than messages that are designed in a relatively simple fashion (e.g., motivational quotes or influencer posts [84,85]). So, if an individual pays attention to a specific stimulus, the brain is involved in the processing of this particular information and binds resources that are not available to process other stimuli. The difficulty of paying attention to several things at once is explained by the limited capacity of the brain’s mental resources, and might also serve to explain why comics are received as less favorable. Of course, additional research is needed to test this assumption. 

Another aspect that is worth mentioning is that, when looking at respondents’ gender, women usually respond more favorable to message content, regardless of its format. So, while preferences for message formats do not vary, females’ more positive responses might be explained by the fact that women share their feelings more readily with more people and in wider social contexts [86,87]. Hence, they might also identify with mental health content to a larger extent. Nevertheless, additional studies have to be conducted in order to provide an answer to this research question. 

## 6. Limitations and Directions for Future Research

The present contribution tested how different forms of content as posted by mental health influencers on Instagram were evaluated by young adults, who constitute the largest user base on Instagram and also reported increasing incidences of mental health problems during COVID-19. The findings from this study might be descriptive, but can serve as an important starting point to develop evidence-based ways to reach the younger segments of the population, whose mental health has been particularly affected by the pandemic [2,3]. Meanwhile, the study presented herein is innovative in that it investigated the responses to different content formats and addressed a very current topic (i.e., mental health). 

Nonetheless, there are several limitations to the study. First, a limitation of the present quantitative survey concerns its reliance on self-reported measures, which present only snapshot results in time [15]. Second, only specific content formats were chosen and can be expanded by more contemporary forms, such as Instagram reels or stories. Third, we also looked at different forms of social support as offered by mental health influencers; however, the forms of support can also be extended in future studies. At the same time, the downsides of mental health content distributed via Instagram must not be ignored, as Instagram use has been associated with several negative effects, such as the comparison of social status [17]. Moreover, the results might be subject to the respondents’ age, and socio-demographic status.

## Data Availability

The data presented in this study are available on request from the corresponding author.

## References

[1] Lee S.A., Jobe M.C., Mathis A.A., Gibbons J.A. (2020). Incremental validity of coronaphobia: Coronavirus anxiety explains depression, generalized anxiety, and death anxiety. J. Anxiety Disord..

[2] Richter F. (2021). Pandemic Causes Spike in Anxiety & Depression. https://www.statista.com/chart/21878/impact-of-coronavirus-pandemic-on-mental-health/.

[3] Stewart C. (2020). What Impact, If Any, Has the Last Few Weeks of the Coronavirus Pandemic Had on Your Mental Health?. https://www.statista.com/statistics/1113551/coronavirus-situation-impact-on-mental-health-uk/.

[4] Pieh C., Budimir S., Probst T. (2020). The effect of age, gender, income, work, and physical activity on mental health during coronavirus disease (COVID-19) lockdown in Austria. J. Psychosom. Res..

[5] Dlugosz P. (2021). Factors influencing mental health among American youth in the time of the Covid-19 pandemic. Personal. Individ. Differ..

[6] Lucibello K.M., Vani M.F., Koulanova A., deJonge M.L., Ashdown-Franks G., Sabiston C.M. (2021). #Quarantine15: A content analysis of Instagram posts during COVID-19. Body Image.

[7] Naslund J.A., Bondre A., Torous J. (2020). Social Media and Mental Health: Benefits, Risks, and Opportunities for Research and Practice. J. Technol. Behav. Sci..

[8] Sadagheyani H.E., Tatari F. (2021). Investigating the role of social media on mental health. Ment. Health Soc. Incl..

[9] Chou W.S., Hunt Y.M., Beckjord E.B., Moser R.P., Hesse B.W. (2009). Social Media Use in the United States: Implications for Health Communication. J. Med. Internet Res..

[10] Bhatt S. (2020). The Rise of Mental Health Influencers. The Economic Times. Indiatimes.com.

[11] Ashman R., Patterson A., Kozinets R.V. (2021). Netnography and Design Thinking: Development and Illustration in the Vegan Food Industry. Eur. J. Mark..

[12] Altendorfer L.M. (2019). Influencer in der Digitalen Gesundheitskommunikation. Instagramer, YouTuber und Co. Zwischen Qualität, Ethik und Professionalisierung.

[13] Mowery D., Bryan C., Conway M. (2017). Feature Studies to Inform the Classification of Depressive Symptoms from Twitter Data for Population Health. arXiv.

[14] Berryman C., Ferguson C.J., Negy C. (2018). Social Media Use and Mental Health among Young Adults. Psychiatr. Q..

[15] Guntuku S.C., Yaden D.B., Kern M.L., Unger L.H., Eichstaedt J.C. (2017). Detecting depression and mental illness on social media: An integrative review. Curr. Opin. Psychol..

[16] Muralidhara S., Paul M.J. (2018). #Healthy Selfies: Exploration of Health Topics on Instagram. JMIR Public Health Surveill..

[17] Ahadzadeh A.S., Sharif S.P., Ong F.S. (2017). Self-schema and self-discrepancy mediate the influence of Instagram usage on body image satisfaction among youth. Comput. Hum. Behav..

[18] MacMillan A. (2017). Why Instagram Is the Worst Social Media for Mental Health. https://time.com/4793331/instagram-social-media-mental-health/.

[19] Lorenz T. (2018). Teens Are Being Bullied ‘Constantly’ on Instagram. https://www.theatlantic.com/technology/archive/2018/10/teens-face-relentless-bullying-instagram/572164/.

[20] Ferguson C.J. (2017). Should We Panic about Teens’ Social Media Use?. http://www.houstonchronicle.com/local/gray-matters/article/Should-we-panic-about-teens-social-media-use-11191051.php.

[21] Orben A. (2017). No, It Hasn’t Been Proven that Instagram is Worst for Young Mental Health. We Need to Stop Misleading the Public with Social Media Pseudoscience. https://medium.com/@OrbenAmy/no-it-hasnt-been-proven-that-instagram-is-worst-for-young-mental-health-36894f33c237.

[22] Jelenchick L.A., Eickhoff J.C., Moreno M.A. (2013). “Facebook depression?” social networking site use and depression in older adolescents. J. Adolesc. Health.

[23] Veretilo P., Billick S.B. (2012). Psychiatric illness and facebook: A case report. Psychiatr. Q..

[24] Simoncic T.E., Kuhlman K.R., Vargas I., Houchins S., Lopez-Duran N.L. (2014). Facebook use and depressive symptomatology: Investigating the role of neuroticism and extraversion in youth. Comput. Hum. Behav..

[25] Reinecke L., Trepte S. (2014). Authenticity and well-being on social network sites: A two-wave longitudinal study on the effects of online authenticity and the positivity bias in SNS communication. Comput. Hum. Behav..

[26] Grieve R., Watkinson J. (2016). The psychological benefits of being authentic on Facebook. Cyberpsychology Behav. Soc. Netw..

[27] Meier A., Schäfer S. (2018). Positive Side of Social Comparison on Social Network Sites: How Envy Can Drive Inspiration on Instagram. Cyberpsychology Behav. Soc. Netw..

[28] Seabrook E.M., Kern M.L., Rickard N.S. (2016). Social Networking Sites, Depression, and Anxiety: A Systematic Review. JMIR Mental Health.

[29] Firsching J. (2020). Instagram Trends 2020: Instagram Überholt Facebook & Karussell-Postings Mit Den Meisten Interaktionen. https://www.futurebiz.de/artikel/instagram-trends-2020/.

[30] Vraga E.K., Bode L., Troller-Renfree S. (2016). Beyond selfreports: Using eye tracking to measure topic and style differences in attention to social media content. Commun. Methods Meas..

[31] Agrawal A.J. (2016). Why Influencer Marketing Will Explode in 2017. https://www.forbes.com/sites/ajagrawal/2016/12/27/why-influencer-marketingwill-explode-in-2017/#3bfaf85c20a9.

[32] Lou C., Yuan S. (2019). Influencer Marketing: How Message Value and Credibility Affect Consumer Trust of Branded Content on Social Media. J. Interact. Advert..

[33] Djafarova E., Rushworth C. (2017). Exploring the Credibility of Online Celebrities’ Instagram Profiles in Influencing the Purchase Decisions of Young Female Users. Comput. Hum. Behav..

[34] Tietjen A. The Mental Health Influencers. http://web.a.ebscohost.com/ehost/pdfviewer/pdfviewer?vid=1&sid=220fa934-9e45-4ca6-8559-3e4ab79372d9%40sdcv-sessmgr02.

[35] Pettigrew S., Donovan R.J., Pescud M., Newton R., Boldy D. (2012). Communicating with older people about positive mental health. J. Public Ment. Health.

[36] McCosker A. (2018). Engaging mental health online: Insights from beyond blue’s forum influencers. New Media Soc..

[37] Davidson L., Chinman M., Sells D., Rowe M. (2006). Peer support among adults with serious mental illness: A report from the field. Schizophr. Bull..

[38] Jarzyna C.L. (2021). Parasocial Interaction, the COVID-19 Quarantine, and Digital Age Media. Hum. Arenas.

[39] Resnick S.G., Rosenheck R.A. (2008). Integrating peer-provided services: A quasi-experimental study of recovery orientation, confidence, and empowerment. Psychiatr. Serv..

[40] Walker G., Bryant W. (2013). Peer support in adult mental health services: A meta synthesis of qualitative findings. Psychiatr. Rehabil. J..

[41] Naslund J.A., Grande S.W., Aschbrenner K.A., Elwyn G. (2014). Naturally occurring peer support through social media: The experiences of individuals with severe mental illness using YouTube. PLoS ONE.

[42] Mead S., Hilton D., Curtis L. (2001). Peer support: A theoretical perspective. Psychiatr. Rehabil. J..

[43] Chang H.J. (2009). Online supportive interactions: Using a network approach to examine communication patterns within a psychosis social support group in Taiwan. J. Am. Soc. Inf. Sci. Technol..

[44] Collarby (2019). 5 Influencer Marketing Trends We Predict for 2020. https://www.collabary.com/blog/influencer-marketing-trends-2020/.

[45] Cilandro A., Graham J. (2020). Studying Instagram beyond Selfies. Social Media + Society April–June: 1–7. https://journals.sagepub.com/doi/pdf/10.1177/2056305120924779.

[46] Saboia I., Pisco Almeida A.M., Sousa P., Pernencar C. (2018). I am with you: A netnographic analysis of the Instagram opinion leaders on eating behavior change. Procedia Comput. Sci..

[47] Dodds A., Chamberlain K. (2017). The problematic messages of nutritional discourse: A case-based critical media analysis. Appetite.

[48] Slater A., Varsani N., Diedrichs P.C. (2017). #fitspo or #loveyourself? The impact of fitspiration and self-compassion Instagram images on women’s body image, self-compassion, and mood. Body Image.

[49] Fox S., Duggan M. (2013). Health Online 2013 [Internet].

[50] Sarasohn-Kahn J. (2008). The Wisdom of Patients: Health Care Meets Online Social Media.

[51] Kearns C., Kearns N. (2020). The role of comics in public health communication during the COVID-19 pandemic. J. Vis. Commun. Med..

[52] Brueck H., Medaris Miller A., Feder S. (2020). China Took at Least 12 Strict Measures to Control the Coronavirus. They Could Work for the US, But Would Likely be Impossible to Implement. https://www.businessinsider.com.au/chinas-coronavirus-quarantines-other-countries-arent-ready-2020-3?r=US&IR=T.

[53] Simpson W. (2018). Feelings in the Gutter: Opportunities for Emotional Engagement in Comics. http://imagetext.english.ufl.edu/archives/v10_1/simpson/#eleven.

[54] Alemany-Pagès M., Azul A.M., Ramalho-Santos J. (2021). The use of comics to promote health awareness: A template using nonalcoholic fatty liver disease. Eur. J. Clin. Investig..

[55] Anand T., Kishore J., Ingle G.K., Grover S. (2018). Perception about use of comics in medical and nursing education among students in health professions’ schools in New Delhi. Educ. Health.

[56] Aleixo P., Sumner K. (2017). Memory for biopsychology material presented in comic book format. J. Graph. Nov. Comics.

[57] Branscum P., Sharma M., Wang L.L., Wilson B., Rojas-Guyler L. (2013). A process evaluation of a social cognitive theory-based childhood obesity prevention intervention: The comics for health program. Health Promot. Pract..

[58] Tversky A., Kahneman D. (1973). Availability: A heuristic for judging frequency and probability. Cogn Psychol.

[59] Locke K.D., Križan Z., Gibbons F.X. (2014). Agency and communion in social comparisons. Communal Functions of Social Comparison.

[60] McNicol N. (2014). Humanising illness: Presenting health information in educational comics. Med. Humanit..

[61] Dobbins S. (2016). Comics in public health: The sociocultural and cognitive influence of narrative on health behaviors. J. Graph. Nov. Comics.

[62] Goodwin J., Tajjudin I. (2016). “What do you think I am? Crazy?”: The Joker and stigmatizing representations of mental ill-health. J. Pop. Cult..

[63] Farthing A., Priego E. (2016). ‘Graphic Medicine’ as a mental health information resource: Insights from comics producers. Comics Grid J. Comics Scholarsh..

[64] Rogers E.M. (2003). Diffusion of Innovations.

[65] Souza F., de las Casas D., Flores V., Youn S., Cha M., Quercia D., Almeida V. Dawn of the Selfie Era: The Whos, Wheres, and Hows of Selfies on Instagram. Proceedings of the 2015 ACM on Conference on Online Social Networks.

[66] WordStream 33 Mind-Boggling Instagram Stats & Facts for 2018. https://www.wordstream.com/blog/ws/2017/04/20/instagram-statistics.

[67] Mathur L.K., Mathur I., Ranga N. (1997). The wealth effects associated with a celebrity endorser: The Michael Jordan phenomenon. J. Advert. Res..

[68] Nicolau J., Santa-María M. (2013). “Celebrity Endorsers” Performance on the “Ground” and on the “Floor”.

[69] Statista (2021). Instagram-Nutzerzahlen Für Österreich Bis 2021. Altersverteilung. Statista..

[70] Statista (2021). Altersverteilung der Österreichischen Instagram-Nutzer im April 2021. https://de.statista.com/statistik/daten/studie/512308/umfrage/instagram-nutzerzerzahlenfuer-oesterreich-nach-alter/.

[71] Broom A., Hand K., Tovey P. (2009). The role of gender, environment and individual biography in shaping quali-tative interview data. Int. J. Soc. Res. Methodol. Theory Pract..

[72] Ellison N.B., Steinfield C., Lampe C. (2007). The benefits of Facebook “friends:” Social capital and college students’ use of online social network sites. J. Comput. Mediat. Commun..

[73] Li F., Bai X., Wang Y. (2013). The Scale of Positive and Negative Experience (SPANE): Psychometric properties and normative data in a large Chinese sample. PLoS ONE.

[74] Jenkins-Guarnieri M.A., Wright S.L., Johnson B. (2013). Development and validation of a social media use integration scale. Psychol. Pop. Media Cult..

[75] Maree T. (2017). The Social Media Use Integration Scale: Toward Reliability and Validity. Int. J. Hum.–Comput. Interact..

[76] Diener E., Wirtz D., William T., Kim-Prieto C., Dongwon C., Oishi S., Biswas-Diener R. (2010). New well-being measures: Short scales to assess flourishing and positive and negative feelings. Soc. Indic. Res..

[77] Schneider S., Schupp J. (2011). The Social Comparison Scale: Testing the Validity, Reliability, and Applicability of the Iowa-Netherlands Comparison Orientation Measure (INCOM) on the German Population. https://papers.ssrn.com/sol3/papers.cfm?abstract_id=1772742.

[78] Nick E.A., Cole D.A., Cho S.J., Smith D.K., Carter T.G., Zelkowitz R.L. (2018). The Online Social Support Scale: Measure development and validation. Psychol. Assess..

[79] MacKenzie S.B., Lutz R.J. (1989). An empirical examination of the structural antecedents of attitude toward the ad in an advertising pretesting context. J. Mark..

[80] Whiting A., Williams D. (2013). Why people use social media: A uses and gratifications approach. Qual. Mark. Res..

[81] Stollfuß S. (2020). Communitainment on Instagram: Fitness Content and Community-Driven Communication as Social Media Entertainment. SAGE Open.

[82] Alkureishi M.A., Johnson T., Nichols J., Dhodapkar M., Czerwiec M.K., Wroblewski K., Arora V.M., Lee W.W. (2021). Impact of an Educational Comic to Enhance Patient-Physician-Electronic Health Record Engagement: Prospective Observational Study. JMIR Hum. Factors.

[83] Craik F.I.M. (2002). Levels of processing: Past, present… and future?. Memory.

[84] Lang A., Park B., Sanders-Jackson A.N., Wilson B.D., Wang Z. (2007). Cognition and emotion in TV message processing: How valence, arousing content, structural complexity, and information density affect the availability of cognitive resources. Media Psychol..

[85] Bradley S.D. (2007). Dynamic, embodied, limited-capacity attention and memory: Modeling cognitive processing of mediated stimuli. Media Psychol..

[86] An J., Weber I. (2016). # greysanatomy vs. # yankees: Demographics and hashtag use on Twitter. arXiv.

[87] Daantje D., Agneta H.F., Arjan E.R.B. (2008). The role of emotion in computer mediated communication. Comput. Hum. Behav..

